# C-reactive protein as a predictor of disease in smokers and former smokers: a review

**DOI:** 10.1111/j.1742-1241.2009.02179.x

**Published:** 2009-11

**Authors:** S Tonstad, J L Cowan

**Affiliations:** 1 School of Medicine, Loma Linda UniversityLoma Linda, CA, USA; 2 UBC Scientific Solutions, Envision HouseHorsham, West Sussex, UK

## Abstract

**Background:**

Cigarette smoking is a classical and a major risk factor in the development of several diseases with an inflammatory component, including cardiovascular disease and chronic obstructive pulmonary disease. Improvements in assays for protein markers of inflammation have led to many studies on these factors and their roles in disease.

**Aims:**

C-reactive protein (CRP) is one such marker and this review focuses on the evidence for using CRP as a diagnostic marker and how levels of this protein are modified according to the smoking status of the patient, both in terms of the current amount of cigarettes smoked and how CRP levels change following smoking cessation.

**Conclusions:**

Assay of CRP levels may be useful in monitoring disease progression and determining risk of future cardiovascular complications. However, as this marker is also an indicator of acute inflammation and challenges to the immune system, some caution must be exercised in interpreting the available data on CRP levels in patients with different chronic comorbidities.

**Review Criteria**

Literature pertaining to C-reactive protein assay in chronic diseases in which smoking is a contributory factor, e.g. coronary heart disease and chronic obstructive pulmonary disease, was compiled from the PubMed and ISI databases.

**Message for the Clinic**

The high-sensitivity test for C-reactive protein is an assay that is commonly used to determine levels of infection, but may also be used to measure levels of underlying inflammation. Smoking may increase circulating levels of CRP, and cessation may cause a reduction. However, this one assay alone may not be enough to predict future disease risk as results could be confounded by other smoking-related comorbidities.

## Introduction

The links between smoking and increased morbidity and mortality have been long established, and current trends indicate that of the one billion smokers worldwide, 500 million will die prematurely from smoking-related diseases (1). Smoking has been shown to have harmful effects on numerous organs of the body and the list of diseases where smoking has been recognised as a contributory factor is extensive (2). It has long been accepted that cigarette smoking is a classical and major risk factor in the development of cardiovascular disease (CVD) and atherosclerosis (3,4). More recently, it has been recognised that CVD contains a component of inflammation and has even been referred to as an inflammatory disease (5,6). In addition, a link has been established between several other chronic inflammatory diseases and smoking, including chronic obstructive pulmonary disease (COPD) (7), rheumatoid arthritis, systemic lupus erythematosus (8) and Crohn’s disease (9). Although the mechanisms linking smoking to these diseases are not well understood, interest in the relationship between inflammatory markers and smoking has been gathering pace in an attempt to provide explanations for smoking-mediated morbidity and mortality.

One such inflammatory marker, C-reactive protein (CRP), may be easily and sensitively measured in a variety of clinical situations to monitor disease progression (10). This review will discuss the relationship between smoking behaviour and levels of CRP, focusing on the use of CRP measurement to predict long-term health in smokers and the outcome of smoking cessation on CRP levels.

## CRP as a marker for acute and chronic inflammation

**Figure 1:**
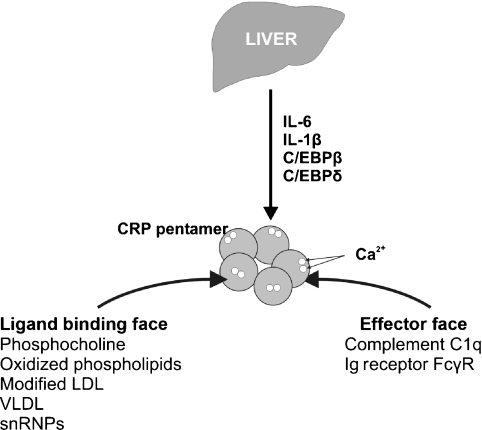
C-reactive protein (CRP) is expressed primarily in hepatocytes, but can be expressed by other cell types. Transcription of CRP mRNA is mediated by interleukins-1β and -6 and transcription factors C/EBPβ and C/EBPδ. Once translated, the mature CRP protein forms a pentamer, characteristic of the pentraxin family of proteins. The CRP pentamer has two binding faces: the ligand binding face binds phospholipids (primarily phosphocholine) in the presence of calcium ions and may also bind snRNPs; the effector face of the pentamer binds substrates including the complement C1q protein and the Ig receptor FcγR. C/EBP, CCAAT/enhancer Binding Protein; LDL, low-density lipoprotein; snRNP, small nuclear ribonucleoprotein; VLDL, very low-density lipoprotein

C-reactive protein is an acute phase plasma protein, synthesised in response to general inflammatory episodes within the body (11,12). It is produced principally by hepatocytes as indicated in Figure 1, but can also be expressed by adipocytes (13) and cultured coronary artery smooth muscle cells (14), suggesting that localised inflammation can induce CRP expression. Indeed, CRP has been detected by immunofluorescence in atherosclerotic plaques from human coronary arteries (15). The acute inflammatory response is induced by numerous challenges to the body, including infections and trauma, and leads to gross changes in the levels of CRP and other acute phase proteins. The primary regulators of CRP and the acute phase proteins are the cytokines interleukin (IL)-6 and IL-1β and tumour necrosis factor (TNF)-α, which are secreted by neutrophil granulocytes and macrophages at sites of injury. These cytokines bind to cell surface receptors and initiate an intracellular signalling cascade, which leads to the activation of several transcription factors. C/EBPβ, a member of the CCAAT-enhancer binding protein transcription factor family, is directly responsible for inducing the transcription of CRP (11). Recent elegant experiments demonstrated the increased binding of C/EBPβ to the CRP promoter, which is located on the proximal arm of chromosome 1 (region 1q23.2), following administration of IL-6 and IL-1β. This led to an induction of CRP mRNA within 3 h, increasing to a peak after 12 h (16).

Once translated, five 22 kDa monomers of CRP form a pentameric disc structure, characteristic of a family of proteins classified as pentraxins (17). Each CRP molecule can bind to a variety of ligands, but shows the highest affinity for phosphocholine residues. These ligands, either autologous ones such as those present on damaged cell membranes, apoptotic cells and in lipoprotein complexes (e.g. low-density lipoprotein, very low-density lipoprotein) or extrinsic ones presented by microorganisms, are bound by CRP, which becomes further aggregated. These aggregates are then recognised by members of the classical complement pathway (particularly the C1q component), thus activating the clearance of the invading microorganism or damaged cell (12). Following the acute phase response, CRP is cleared from the plasma, with the protein having a half-life of approximately 19 h (18).

As well as the major increases in expression of CRP in response to infection or tissue injury, minor elevation in CRP levels has been recognised as a possible marker of disease in systemic conditions (19). This has been aided by the development of assays to measure CRP levels with far greater sensitivity than previous methods (described as high-sensitivity or hs-CRP assays) and has led to a flood of literature investigating CRP levels in healthy and diseased individuals (12,20).

From numerous studies, a large variation in what is considered to be normal levels of CRP has been described. For example, the American Heart Association defines a serum CRP concentration of < 1.0 mg/l as being a low risk for developing CVD and a measurement of > 3.0 mg/l as being a high risk. These values are in contrast to those observed during acute activation of CRP in response to infection or inflammatory disease, when CRP levels are > 10 mg/l and can become > 500 mg/l (21), although such extreme elevations in CRP levels are most likely as a result of infection (e.g. 88% of patients in a study of 130 patients with CRP > 500 mg/l (22)). However, despite the numerous studies that describe the use of serum CRP levels as a predictive marker for determining risk of CVD, more recent meta-analyses appear to downplay the significance of CRP as a prognostic marker (21,23,24).

## Do CRP levels vary according to smoking status?

In recent years, there has been a large volume of studies, some of which are conflicting, in which serum CRP concentrations have been measured in parallel to smoking status because of the possible link between smoking and the induction of inflammatory pathways (25).

Smokers have increased numbers of white blood cells, mainly because of a particular increase in polymorphonuclear neutrophils, which are released from the bone marrow and recruited to inflamed tissue (26). IL-β and IL-6, which are increased in response to lung inflammation and are implicated in the induction of CRP gene expression, may mediate the stimulation of bone marrow cells (27). In one study, levels of inflammatory markers were measured in the bloodstream of intermittent smokers 24 h after they had two cigarettes following 9 days of abstinence. TNF-α, IL-10 and IL-1β did not change, but levels of IL-8 increased after 3 h (28).

In one of the earlier studies of CRP levels in smokers, and before the advent of assays with higher sensitivities, CRP was found to be significantly higher in male and female smokers compared with non-smokers (median values of 1.0 mg/l and 11.2 mg/l for male non-smokers and smokers, respectively, and for females 2.0 mg/l and 11.6 mg/l, respectively) (29). Such strikingly different values have not been observed in more recent studies. Furthermore, more than half (35/60) of the smoking cohort from this initial study had CRP values > 10 mg/l, which could be considered as reflecting an inflammatory episode (21).

The complexity of cytokine-mediated inflammation is highlighted by a study showing that although smoking status did correlate with a significant elevation in levels of IL-6 and serum amyloid protein A, another acute phase protein, the increase in CRP levels observed in smokers was not found to be statistically significant (30). Another larger study found that mean CRP levels were significantly lower in never-smokers (p < 0.0001) than in current smokers (31). The CRP assays from these studies and others mentioned in this text are summarised in Table 1.

**Table 1:** Summary of CRP assays according to smoking status in different studies

References	Population studied	No. of subjects for each group	CRP levels (mg/l) in current smokers (or subgroups)	CRP levels (mg/l) in former smokers (or subgroups)	CRP levels (mg/l) in never or non-smokers	Significant difference between current smokers and never- or non-smokers p-value
Helmersson et al. (30)	642 Swedish men, age 77 years	Current 55 Former 391 Never 196	1.65	1.8	2.31	Not significant
Wannamethee et al. (31)	2920 British men, age 60–79 years	Current 391 Former 1503 Never 873	2.53 (95% CI 2.27–2.80)	1.58 (95% CI 1.49–1.66)	1.35 (95% CI 1.26–1.46)	< 0*.*0001
Lowe et al. (32)	1690 British men, age 49–67 years	Current 536 Former 744 Never 272	No. smoked/day 1–14 = 1.87 15–24 = 2.32 > 25 = 2.05	Time since quit > 10 years = 1.36 5–9 years = 1.34 1–4 years = 1.66 < 1 years = 2.10	1.13	< 0*.*001 (lightest smokers vs. never-smokers) 0*.*037 (> 10 year quitters vs. never-smokers
Oshawa et al. (33)	1926 Japanese men, age 40–69 years	Non 661 Former 503 Current 760	0.98 (SD = 1.30)	0.87 (SD = 1.24)	0.79 (SD = 1.20)	< 0*.*01
Fröhlich et al. (34)	2305 men from Augsburg, Germany, age 25–74 years 2211 women from Augsburg, Germany, age 25–74 years	Not shown Not shown	Regular 1.92 Occasional 1.41 Regular 1.52 Occasional 1.15	1.27 1.39	1.03 1.41	Not shown Not shown
Bermudez et al. (35)	340 US women, mean age 60.1 years	Non 43.4% Former 28.6% Current 28.0%	0.38 (IQR 0.18–0.83)	Not shown	0.30 (IQR 0.13–0.57)	0*.*032

CI, confidence interval; CRP, C-reactive protein; IQR, interquartile range; SD, standard deviation.

A dose-dependent correlation between CRP and smoking habits was demonstrated in the ‘Speedwell’ survey of British men. CRP levels were increased from 1.13 mg/l in never-smokers to 1.87, 2.32 and 2.05 mg/l in those who smoked 1–14, 15–24 and > 25 cigarettes, respectively per day (32). However, another study conducted in people of Japanese ethnicity (the Iwate-Kenpoku Cohort study) failed to identify any significant relationship between serum CRP concentration and the number of cigarettes smoked per day (33).

## Effect of gender on smoking and CRP status

In one of the few studies that examined gender-specific differences for smoking and CRP levels, the MONICA study from Germany, serum CRP concentrations were significantly higher in male regular smokers than male never-smokers (1.92 mg/l vs. 1.03 mg/l, p < 0.001), but no significant difference was observed in women (1.52 mg/l for regular smokers vs. 1.41 mg/l for never-smokers) (34).

This gender difference has been observed previously (35), and the authors of the MONICA study suggest that the difference in CRP levels may be because of differing ‘puffing behaviour’ in women. Women tend to smoke less non-filtered cigarettes, smoke more low-yield cigarettes and take smaller and shorter puffs.

However, a more recent study takes into account whether women were taking hormones orally (36). In this case, a correlation between smoking status and increased serum CRP concentrations was indeed observed, but only in those who were not taking oral hormones, suggesting that such treatments could be masking changes in CRP levels in the female population.

## CRP status in smokers with chronic diseases

The focus of most of the research attempting to link CRP expression and incidence of chronic diseases that are confounded by smoking status has been in the area of coronary heart disease (CHD), and a recent review provides a comprehensive analysis of this field (10). Furthermore, the formation of atherosclerotic plaques in response to elevated CRP levels and hence the increased risk of atherothrombosis because of plaque rupture have been discussed in great detail by other authors (37,38), and the numerous other pathways and mechanisms by which cigarette smoking can induce inflammation and therefore lead to plaque formation are also reviewed elsewhere (39). For example, work detailing postmortem analysis of coronary arteries in smokers vs. non-smokers who died between the ages of 15 and 34 as a result of external factors (40) showed that advanced (grade 5) atherosclerotic lesions were far more prevalent in smokers compared with non-smokers [Odds ratio (OR) 9.61, 95% confidence interval (CI) 2.34–39.57]. Although assay of CRP in these cadavers showed no correlation between smoking status and increased CRP (41), suggesting plaque formation cannot be simply explained by a single factor, at least in younger smokers.

About 20% of smokers suffer from COPD, and one study demonstrated that CRP levels correlated with pack-years of smoking and predict all-cause mortality in patients with mild to moderate COPD in the short-term, although this prediction becomes weaker with time (42). However, in patients with moderate to very severe COPD, no relationship could be identified between CRP levels and likelihood of survival (43).

Although a relationship between increased CRP and deaths from respiratory disease has not been comprehensively proven, high CRP is associated with a yearly decrease in forced expiration volume of sustained smokers. The authors therefore suggested that combining the results of the CRP assay and forced expiration volume would help predict outcome, and thus enable early intervention (42).

In contrast, another study examining CRP levels in patients with moderate to severe COPD reported that, although there was a significant difference in CRP levels in the COPD patients, there was no difference in CRP status between control groups of smokers and non-smokers (44).

In a study of patients with different forms of angina, the serum concentration of CRP did not correlate with the type of angina exhibited by the patient, but did significantly correlate with the patient’s smoking status (p < 0*.*0001) (45).

Many studies have examined CRP levels in cancer patients compared with healthy controls and have identified a relationship between raised CRP and disease state (46). However, systematic review of the data from these studies by these authors suggests that the majority of studies fail to provide adequate statistical analyses and also fail to take into account many confounding factors, with the majority of studies being retrospective (and hence at risk of introducing bias) (47). A further independent study (48) demonstrated an increased likelihood of cancer incidence (OR: 1.3; 95% CI: 1.0, 1.6), when comparing those with a baseline CRP level of < 1 mg/l, with those > 3 mg/l, although this interaction was not significant (p = 0.06). However, both studies showed a significant positive correlation between raised CRP and risk of developing lung and colorectal cancer. Although smoking status of the subjects was considered, no analysis of whether this was a confounding factor in cancer risk was presented.

In these analyses it is still not clear whether the elevation of CRP is a cause or consequence of tumour growth, although one study did take this into account (48), and demonstrated that the mean time between blood sampling for assay of CRP and development of cancer was 5.8 years, suggesting the former. However, if those cases of cancer that were diagnosed within 2 years of sampling (hence cancers that may have been present, but not yet diagnosed) were removed from the analysis, the relationship between elevated CRP levels and increased risk of any cancer no longer existed. It is entirely possible that underlying occult tumours in any subjects discussed in this review may be influencing CRP levels. Without similar long-term longitudinal studies, such a confounding factor will not become apparent.

## Serial assay of CRP levels

Of possible significance to the previously discussed variability is the fact that many of these studies examining CRP levels in smokers measure the concentration of this biomarker at a single point in time. Given the massive induction of CRP in response to acute inflammation or infection (up to and exceeding 500 mg/l) and the half-life of the protein in the circulation (19 h), it is feasible that CRP levels may remain over a significant level for a week after the initial elevation.

When serum concentrations of CRP in male and female smokers were measured on five independent occasions over a 6-week study, they were found to differ significantly over the whole study group (median 2.7 mg/l in smokers vs. 1.2 mg/l in non-smokers, p = 0*.*05), but these differences were not significant when the individual results from each gender were examined (49). As this study examined CRP on multiple occasions, the variability of serum levels in subjects could be calculated, with the within-subject CRP values varying by more than 100% of the standard deviation for both smokers and non-smokers, and the between-subject variability being even higher. The authors made the salient point that this could be because of CRP levels being a marker of even the mildest levels of inflammation, such as from a sporadic mild acute illness. They did note, however, that women with consistently higher CRP levels were all taking oral contraceptives containing oestrogen, which has been found to correlate significantly with elevated CRP levels (50,51).

## CRP and early and indirect exposure to tobacco smoke

In addition to the reports observing CRP levels in long-term smokers, studies of adolescent smokers have also reported significantly higher levels of CRP. When data from a study of adolescents were analysed, an increase in CRP levels was observed, with heavy smokers having twice the CRP concentration compared with non-smokers (52). Furthermore, CRP levels are significantly increased in children who are exposed to secondhand smoke (53).

However, contradicting results have been observed in never-smoking adults exposed to secondhand smoke with one group reporting an increase in CRP levels (54), while a second study did not observe a significant change in CRP status, even though other biomarkers of CVD risk (fibrinogen, homocysteine) were increased (55).

## CRP levels following smoking cessation

Most studies that have examined CRP status in former smokers suggest that levels fail to fall immediately upon cessation, which reflects the fact that the underlying tissue damage caused by smoking takes some time to recover (25).

In adults without CHD, CRP levels were significantly higher for current smokers compared with ex-smokers (median 1.9 mg/l vs. 1.6 mg/l, p < 0*.*001) and never-smokers (median 1.1 mg/l, p < 0*.*001). In addition, CRP levels remained significantly higher than in never-smokers for up to 5 years following cessation (p < 0*.*001) (56). Similarly, another study demonstrated that current smokers had higher levels of CRP compared with never-smokers (2.53 mg/l vs. 1.35 mg/l, p < 0*.*0001) and former cigarette smokers (1.58 mg/l, p < 0*.*0001), with the difference in CRP serum concentration remaining significant between never-smokers and former smokers after 5 years following quitting (31). Furthermore, CRP levels were reduced to those observed in never-smokers only in those participants who had quit for over 20 years.

In the ‘Speedwell’ survey, for those who had quit smoking for less than a year, CRP levels remained at 2.10 mg/l, but subsequently reduced with time to 1.34 mg/l 5–9 years and 1.36 mg/l > 10 years after quitting, with this level of CRP still higher than that of the never-smokers (32).

A study by Fröhlich et al. (34) has one of the longest follow-up periods, with levels of CRP that were as low as a mean of 1.25 mg/l following 30–55 years of cessation in male subjects, compared with 1.92 mg/l in regular smokers.

As before, most of these surveys only consider CRP levels at a single point in time, but serial studies following CRP levels before and after smoking cessation have also been undertaken (57,58). When serum CRP levels were compared between a group of continuing smokers and a group who had successfully abstained from smoking for a year following 8 weeks of transdermal nicotine patch therapy, the levels of CRP were not significantly different from those that were measured the year before (57). Even though CRP levels did rise slightly in continuing smokers and fell slightly in those who had quit for a year, the total difference in change from the mean baseline CRP levels between the continuing smokers and the quitters was only 0.66 mg/l (p = 0*.*26). Furthermore, CRP levels did not significantly decrease in a cohort of female smokers following 6 weeks of smoking cessation with nicotine replacement therapy (58).

Similarly, no reduction in CRP levels was observed in smokers who had reduced their cigarette intake by at least 50% in the 6 months after a baseline CRP reading was taken (59). Clearly, following the CRP status of these subjects for a number of years would confirm how long it may take for CRP levels to normalise following smoking cessation.

## Genetic factors that influence serum CRP levels

Recent studies have demonstrated that CRP levels are also influenced by genetic factors, with heritability for raised CRP ranging from 39% to 52% in family and twin studies (60).

CRP is transcribed from chromosome 1, and the ∼2 kb mRNA that encodes the 22 kDa protein contains two exons, with the single intron having a polymorphic GT dinucleotide repeat, which varies in length from 9 to 25 copies (61). The number of these microsatellite repeats varies between races, with the three most common genotypes GT^16/16^, GT^16/21^ and GT^21/21^ found in 78% of Caucasians, but in only 20% of African-Americans (61). These genotypes are associated with low CRP levels but, in contrast, individuals with a GT^18/18^ or GT^20/20^ genotype have significantly higher serum CRP, although the mechanisms underlying this change in expression have yet to be elucidated. In addition, there have been at least 40 single nucleotide polymorphisms (SNPs) identified in the CRP gene locus (60), although only eight of the SNPs have been identified to occur with > 5% frequency (62) with the majority of SNPs found in the 3′ untranslated region or the promoter. This latter observation suggests that upstream mediators of CRP expression may be responsible for differing effects on the genetic variants. For those SNPs found in the 3′ untranslated region, they could be exerting differential effects on the stability of the message, or even altering binding sites for microRNAs (miRNA), which could in turn affect the translational regulation of the mRNA. To date, no reports of miRNA regulation of CRP expression have been published, but miR-120 has been shown to be involved in the translational repression of pentraxin A1 (63).

It is perhaps disappointing that, despite the huge amount of data being generated on the disease risks associated with certain polymorphisms and CRP levels, no direct link has yet been established as to whether any of the known polymorphisms in the CRP gene and their effect on CRP status is confounded by the smoking status of the subject.

## Conclusions

Since the development of high-sensitivity CRP assays, this acute phase protein has been examined in a large number of studies, and increased CRP has been found to be associated with increasing age, body mass index, weight and reduced fitness levels. Significant differences in CRP levels relative to socioeconomic position, ethnicity and gender have also been reported (12,20).

These results are in addition to the numerous studies that examine CRP in patients with CVD and many other diseases in which smoking is a known aggravating factor. However, as CRP levels can change so rapidly in response to even the most minor of challenges to the immune system and also to tissue injury, several authors have cautioned against the view that CRP can be used as a prognostic marker in assessing future disease risk (21,23,24).

The ambitious Emerging Risk Factors Collaboration is currently in the process of collecting data on circulating lipid factors and markers of inflammation, including CRP, from over 1.1 million participants (64). This meta-analysis may finally be able to determine whether there is a definitive relationship between smoking status and CRP levels and may also be able to distinguish whether CRP is itself a causal risk factor in CVD or a marker reflecting the status of other cardiovascular risk factors (10). Measurement of CRP levels before and following smoking cessation may be a useful indicator of any decrease in risk of CVD or COPD for the patient, and therefore encourage long-term abstinence. If such assays were undertaken, however, it would be important to consider on a case-by-case basis whether any acute confounding events precipitated the attempt at smoking cessation [e.g. myocardial infarction (MI)]. In these cases, the precipitating event would itself cause an initial elevation in CRP levels and hence be inappropriate as a measure of encouragement.
